# Assessing Response to Difelikefalin in Adults Receiving Haemodialysis: A Multidisciplinary, Person‐Centred Framework for Chronic Kidney Disease‐Associated Pruritus

**DOI:** 10.1111/jorc.70091

**Published:** 2026-11-11

**Authors:** Lucas Maciel de Almeida Corrêa, Isabela Malerba Pinheiro, Yan Roberth Delmiro Silva, Guilherme Diniz Ferreira

**Affiliations:** ^1^ Faculdade de Medicina de São José do Rio Preto (FAMERP) São José do Rio Preto São Paulo Brazil; ^2^ Pontifícia Universidade Católica de Minas Gerais (PUC Minas) Belo Horizonte Minas Gerais Brazil; ^3^ Universidade Federal de Alagoas (UFAL), Campus Arapiraca Arapiraca Alagoas Brazil; ^4^ Faculdade de Ciências Médicas de Minas Gerais (CMMG) Belo Horizonte Minas Gerais Brazil

**Keywords:** haemodialysis, patient‐reported outcome measures, pruritus, quality of life, sleep

## Abstract

**Background:**

Chronic kidney disease‐associated pruritus remains under‐recognised in adults receiving haemodialysis and is associated with impaired sleep, quality of life and daily functioning. Translating treatment effects into consistent routine monitoring remains challenging.

**Objectives:**

To synthesise evidence relevant to multidimensional assessment of response to difelikefalin and propose a practical, person‐centred framework for haemodialysis care.

**Design:**

State‐of‐the‐art narrative review informed by a structured pragmatic literature search.

**Participants:**

Not applicable.

**Measurements:**

Evidence was synthesised across itch intensity, sleep‐related disruption, pruritus‐related quality of life, wider symptom burden, patient‐reported outcome measures, implementation in kidney care and exploratory biomarkers.

**Results:**

Evidence supports improvements with difelikefalin in itch intensity, sleep‐related disruption and pruritus‐related quality of life. We propose a brief core set combining the Worst Itching Intensity Numerical Rating Scale with sleep and daily‐burden questions, supplemented by Skindex‐10 and 5‐D Itch when indicated. The combined set, brief burden questions and within‐session assessment timing are unvalidated implementation proposals. Full response, partial response and persistent burden are pragmatic communication labels, not validated phenotypes. Reviews around Weeks 4 and 12 are informed by trial assessment points; subsequent 8‐ to 12‐week intervals are pragmatic and unvalidated. Established instrument thresholds support interpretation of change, while exploratory biomarkers remain research‐only.

**Conclusions:**

The proposed person‐centred framework may support multidimensional assessment of difelikefalin response. Its feasibility, response categories and clinical utility require prospective validation before it can be considered a validated clinical pathway.

## 1 Introduction

Chronic kidney disease‐associated pruritus (CKD‐aP) is a frequent and still under‐recognised problem in adults receiving haemodialysis. It should not be viewed as a minor sensory complaint, because it is consistently associated with impaired sleep, reduced quality of life, greater symptom burden and worse patient‐reported outcomes (Rigatto et al. 2024; Buades et al. 2024; Wang et al. 2025; Turgay and Özdemir Eler 2026; Downey et al. 2026). Observational and longitudinal studies have also shown that itch commonly coexists with fatigue, pain, anxiety, depressive symptoms and sleep disturbance, thereby amplifying daily burden and affecting the overall experience of living with kidney disease (Sukul et al. 2021; Rhee et al. 2022; Sukul et al. 2023; Malhotra et al. 2026). For kidney care teams, the practical challenge is therefore not simply to record whether itch is present, but to judge whether treatment is reducing the domains that matter most to the person receiving care.

Evidence supports the benefit of difelikefalin for moderate‐to‐severe CKD‐aP in adults receiving haemodialysis (Fishbane et al. 2020; Topf et al. 2022; Zuo et al. 2026). Subsequent analyses suggest that the clinical effect extends beyond itch intensity alone and includes sleep‐related disruption and pruritus‐related quality of life (Weiner et al. 2024; Ständer et al. 2024; Fotheringham et al. 2024; Sheldon et al. 2026). Even so, average improvements at the group level do not fully explain why some individuals achieve broad relief whereas others continue to report clinically important burden. This review therefore focuses on how response to difelikefalin can be assessed in routine kidney care through a practical, person‐centred and multidimensional framework. The proposed framework, which requires prospective validation, is summarised in Figure 1.

**Figure 1:**
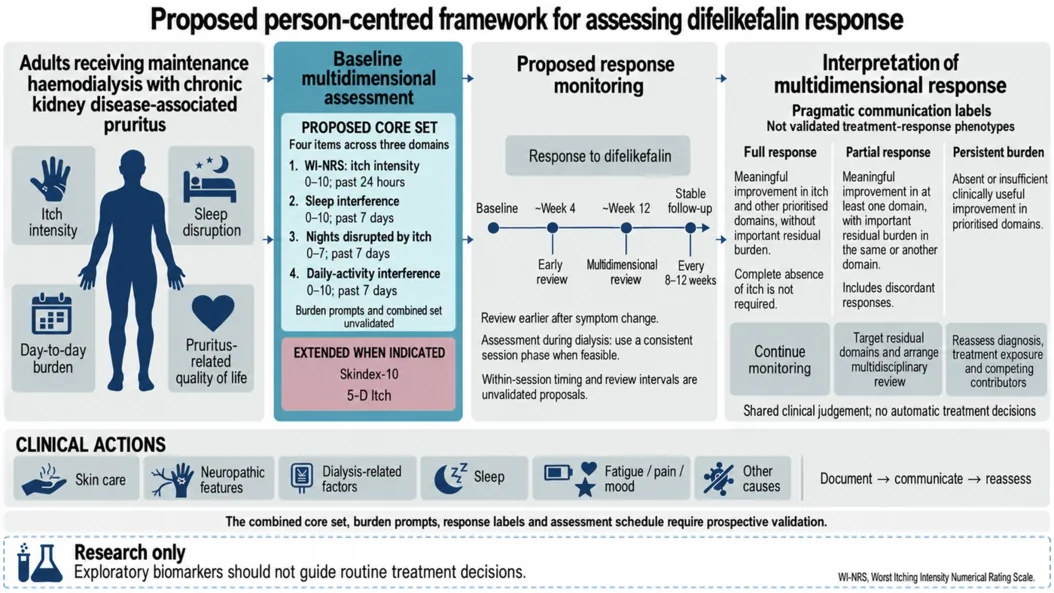
Proposed multidisciplinary, person‐centred framework for assessing difelikefalin response in adults receiving maintenance haemodialysis with chronic kidney disease‐associated pruritus (CKD‐aP). The proposed four‐item core set covers three domains: itch intensity, sleep and daily burden. Skindex‐10 and 5‐D Itch provide extended assessment when indicated. Full response, partial response and persistent burden are pragmatic communication labels, not validated treatment‐response phenotypes. Discordance between domains can be described within partial response. The combined core set, brief burden questions, within‐session assessment timing and follow‐up schedule—including reviews around Weeks 4 and 12 and later 8‐ to 12‐week intervals—are unvalidated implementation proposals. Instrument‐specific meaningful‐change thresholds do not validate these labels or the combined pathway. Residual burden informs domain‐specific review and multidisciplinary discussion, without automatic treatment decisions. Exploratory biomarkers remain research‐only. The framework requires prospective evaluation of feasibility, acceptability, consistency of interpretation and clinical utility. WI‐NRS, Worst Itching Intensity Numerical Rating Scale.

The implementation gap is increasingly important. Recent synthesis identified substantial heterogeneity in patient‐reported outcome measures used in CKD‐aP. Broader dialysis guidance also identifies inconsistent symptom recognition and monitoring (Mehrotra et al. 2023; Bai et al. 2025; Bennett et al. 2025). Earlier EDTNA/ERCA educational work emphasised proactive symptom identification and structured assessment within haemodialysis care, while subsequent multicentre peer‐reviewed data demonstrated that CKD‐aP may remain under‐reported and incompletely recognised in haemodialysis units (Liossatou et al. 2022; Liossatou et al. 2025). Kidney care teams need practical ways to select measures, plan reassessment, interpret discordant symptom trajectories and act on residual burden. This review addresses that evidence‐to‐workflow gap without proposing a new biological classification of treatment response.

## 2 Materials and Methods

This state‐of‐the‐art narrative review addresses assessment of difelikefalin response in adults receiving maintenance haemodialysis, particularly those with moderate‐to‐severe CKD‐aP. Its scope comprises itch intensity, sleep, pruritus‐related quality of life, wider symptom burden, patient‐reported outcome measures, implementation and exploratory biological correlates in routine kidney care.

PubMed, Web of Science, Scopus and Embase were searched without study‐design filters. English‐language publications published from 1 January 2020 through 15 September 2026 were eligible; these limits were applied during selection. Complementary searches addressed responses to difelikefalin and broader symptom assessment, nursing, implementation and biomarkers. Database‐specific strategies are provided in Table S1. Reference lists of key reviews and pivotal studies were checked for directly relevant publications. Eligible peer‐reviewed full‐text evidence included randomised trials, pooled and post hoc analyses, observational and real‐world studies, psychometric studies, systematic reviews, guidance, kidney nursing research and selected mechanistic studies. Selected professional‐society guidance and educational resources informed implementation and role delineation, but not efficacy or response thresholds. Grey literature was not otherwise systematically searched. Paediatric studies, non‐kidney‐related pruritus, non‐English publications, conference abstracts and purely preclinical studies were excluded from support for practice proposals.

Titles and abstracts were screened for relevance to these domains. Full texts were reviewed when they informed a proposed practice statement, or eligibility was uncertain. Selection was iterative and purposive, without duplicate independent screening; directly applicable trials, psychometric and real‐world studies, and relevant syntheses were prioritised. Representative evidence is mapped in Table 1. Findings were integrated qualitatively, with exploratory biomarkers separated from clinical decision‐making. No formal risk‐of‐bias assessment, evidence grading or quantitative pooling was performed. Selection bias, author judgement and heterogeneous evidence limit the synthesis; lower‐level evidence was not used to establish causal or comparative treatment effects.

**Table 1:** Representative evidence informing the proposed multidisciplinary monitoring framework.

Study	Design/setting	Population	Response domains/instruments	Follow‐up	Relevance to framework
Fishbane et al. (2020)	Phase 3 randomised controlled trial	Adults receiving haemodialysis with moderate‐to‐severe chronic kidney disease‐associated pruritus (CKD‐aP)	WI‐NRS; 5‐D Itch; Skindex‐10	12 weeks	Pivotal efficacy evidence using a ≥ 3‐point WI‐NRS responder endpoint
Topf et al. (2022)	Pooled Phase 3 analysis	Adults receiving haemodialysis	WI‐NRS; 5‐D Itch; Skindex‐10	12 weeks	Consistency of multidomain treatment benefit
Weiner et al. (2024)	Post hoc analysis of Phase 3 data	Adults receiving haemodialysis	Itch‐related sleep disruption	12 weeks	Supports explicit assessment of sleep‐related burden
Fotheringham et al. (2024)	Single‐arm intervention study	Adults receiving haemodialysis	WI‐NRS; sleep; Skindex‐10; 5‐D Itch; broader quality of life	12 weeks	Illustrates multidimensional response beyond itch intensity
McCafferty et al. (2025)	Exploratory analysis	Adults receiving maintenance haemodialysis	Baseline itch severity and treatment outcomes	12 weeks	Explores associations between baseline itch severity and treatment outcomes
Bai et al. (2025)	Systematic review	CKD‐aP literature	Patient‐reported outcomes and instruments	Variable	Demonstrates heterogeneity of outcome measurement
Vernon et al. (2021)	Psychometric validation	Adults receiving haemodialysis with moderate‐to‐severe CKD‐aP	WI‐NRS	Trial‐based longitudinal data	Supports a ≥ 3‐point WI‐NRS reduction as a conservative meaningful within‐patient change threshold
Vernon et al. (2026)	Psychometric validation	Adults receiving haemodialysis with moderate‐to‐severe CKD‐aP	Skindex‐10; 5‐D Itch	Trial‐based longitudinal data	Supports ≥ 15‐point Skindex‐10 and ≥ 5‐point 5‐D Itch meaningful‐change thresholds
Bennett et al. (2025)	KDIGO workshop report	People receiving dialysis	Symptom identification, patient‐reported outcomes, monitoring and nursing implementation	Not applicable	Supports structured symptom monitoring and role delineation within multidisciplinary care
Liossatou et al. (2025)	Multicentre kidney nursing survey	Kidney nurses caring for adults receiving haemodialysis	Recognition, reporting and management of CKD‐aP	Cross‐sectional	Supports proactive elicitation and implementation in haemodialysis units
Zuo et al. (2026)	Phase 3 randomised controlled trial	Chinese adults receiving haemodialysis	WI‐NRS; 5‐D Itch; Skindex‐10	12 weeks	Provides efficacy evidence with Weeks 4 and 12 outcome assessments; these timepoints inform, but do not validate, the proposed clinical reassessment schedule
Sheldon et al. (2026)	Systematic review of real‐world evidence	Adults with CKD‐aP receiving difelikefalin	WI‐NRS; sleep; quality of life; safety	Up to 12 months	Documents heterogeneity of real‐world outcome measurement and informs priorities for prospective standardisation
Turgay and Özdemir Eler (2026)	Mixed‐methods study	Adults receiving haemodialysis	Symptom burden and patient experience	Cross‐sectional/qualitative	Reinforces multidimensional burden and person‐centred assessment
Downey et al. (2026)	Cross‐sectional treatment‐experience study	People receiving dialysis	Pruritus severity, treatment experience and burden	Cross‐sectional	Reinforces variability of symptom burden and treatment experience
Spencer et al. (2026)	Retrospective biomarker analysis	Adults with CKD‐aP	Inflammatory biomarkers and response	Biomarker sampling at baseline and Week 12	Research‐only layer; not suitable for routine treatment selection

*Note:* This table maps representative published evidence and study assessment timepoints. It does not establish validity of the author‐proposed combined core set, brief burden questions, communication labels, within‐session assessment timing or clinical reassessment schedule.

Abbreviations: KDIGO, Kidney Disease: Improving Global Outcomes; WI‐NRS, Worst Itching Intensity Numerical Rating Scale

## 3 Results

### 3.1 Clinical Benefit Extends Beyond Itch Intensity

The pivotal trial by Fishbane et al. (2020) established that difelikefalin reduces itch intensity in adults receiving haemodialysis with moderate‐to‐severe CKD‐aP. Pooled analyses and subsequent studies support the consistency of this treatment signal (Topf et al. 2022; Zuo et al. 2026). Improvements have also been reported in itch‐related sleep disruption, pruritus‐related quality of life and broader health‐related quality of life (Weiner et al. 2024; Ständer et al. 2024; Fotheringham et al. 2024; Sheldon et al. 2026). These findings support assessment across several domains. Recent real‐world synthesis also indicates that outcome measurement remains heterogeneous across routine‐care studies, providing a rationale for evaluating a brief, consistent monitoring set in routine care (Sheldon et al. 2026).

### 3.2 What Mean Treatment Effects Can and Cannot Tell Us

Mean group effects are important, but they do not fully capture the variability of response seen in clinical practice. Baseline itch severity informs interpretation of outcomes (McCafferty et al. 2025), but itch improvement can coexist with residual sleep disruption or impaired quality of life. This matters because clinically meaningful improvement may occur without full recovery of sleep, quality of life or associated symptoms. Reviews of the field therefore support the use of multidimensional assessment rather than reliance on a single scale or a single threshold (Wang et al. 2025; Bai et al. 2025; Vernon et al. 2026). Importantly, psychometric validation in adults receiving haemodialysis with moderate‐to‐severe CKD‐aP supports a ≥ 3‐point reduction in the Worst Itching Intensity Numerical Rating Scale (WI‐NRS) as a conservative meaningful within‐patient change threshold, while recognising that smaller improvements may still be meaningful to some individuals (Vernon et al. 2021). This threshold was evaluated using weekly means of daily 24‐h ratings; applying it to isolated chairside ratings is an extrapolation. More recent psychometric work supports reductions of ≥ 15 points in Skindex‐10 and ≥ 5 points in the 5‐D Itch scale as meaningful within‐patient changes (Vernon et al. 2026). These thresholds aid interpretation of change but should not be treated as interchangeable measures of the same construct or as absolute definitions of treatment success. They do not validate the proposed multidimensional response categories.

### 3.3 Biomarkers Remain a Research‐Only Layer

CKD‐aP is biologically heterogeneous and appears to arise from converging immune, neurological, metabolic and cutaneous mechanisms rather than a single pathway (Schricker and Kimmel 2021; Verduzco and Shirazian 2020; Agarwal et al. 2021; Kljajić et al. 2026). Recent mechanistic work has also highlighted possible contributions from uraemic solutes, xerosis, altered opioid signalling, neuropathic factors and disturbances in mineral metabolism (Verduzco and Shirazian 2020; Yang et al. 2023; Kljajić et al. 2026). In a retrospective biomarker analysis, Spencer et al. (2026) reported coordinated changes in selected inflammatory markers among some difelikefalin responders. These findings are scientifically interesting, but they remain exploratory, retrospective and unsuitable for determining treatment access, treatment selection, continuation or discontinuation in current practice. Biomarkers therefore do not form part of the proposed clinical minimum data set and are retained in this review only as a research layer that may inform future prospective validation.

## 4 Discussion

The contribution of this review is not the proposition that CKD‐aP is multidimensional, which is already well established, nor the creation of a new patient‐reported outcome measure or biological response taxonomy. Rather, it addresses an implementation gap between efficacy evidence and routine haemodialysis care. Existing studies use multiple instruments and timepoints, while symptom assessment in dialysis remains incompletely standardised (Rhee et al. 2022; Mehrotra et al. 2023; Bai et al. 2025; Bennett et al. 2025). The framework proposes a brief core assessment, reassessment points, interpretation of instrument‐specific change, documentation and multidisciplinary review of residual burden. Evidence supports the individual validated instruments; the combined assessment, brief burden questions, response labels and implementation schedule remain proposals for prospective evaluation.

We propose three pragmatic communication labels—full response, partial response and persistent burden—which are not validated treatment‐response phenotypes. Full response describes meaningful improvement in itch and in other domains prioritised at baseline, with no clinically important residual burden in those domains; complete absence of itch is not required. Partial response describes meaningful improvement in at least one assessed domain with clinically important residual burden in another or the same domain, including discordant improvement in itch and sleep. Persistent burden describes absent or insufficient clinically useful improvement in the domains prioritised by the person receiving care. These descriptions rely on shared clinical judgement and have no validated classification cut‐offs. Residual burden warrants reassessment of xerosis, neuropathic features, dialysis‐related factors, mineral and bone disorder, and competing causes of itch (Verduzco and Shirazian 2020; Latus et al. 2025; Wang et al. 2025; Bai et al. 2025; Kljajić et al. 2026).

This multidimensional approach is supported by the broader literature on symptom burden in haemodialysis. CKD‐aP often clusters with fatigue, pain, anxiety, depressive symptoms and sleep disruption, and these domains may improve to different degrees over time (Rhee et al. 2022; Sukul et al. 2023; Malhotra et al. 2025; Turgay and Özdemir Eler 2026; Downey et al. 2026). A narrow focus on itch intensity alone can therefore underestimate residual burden, whereas structured follow‐up helps clinicians identify whether a person is sleeping better, coping better and functioning better in daily life (Wang et al. 2025; Bai et al. 2025; Bennett et al. 2025).

The place of biomarkers should remain deliberately separate from routine response monitoring. Current evidence does not justify biomarker‐guided restriction of therapy, nor does it support routine endotype‐based treatment decisions. Instead, exploratory biomarkers are best regarded as research tools that may eventually help explain response heterogeneity or identify pharmacodynamic changes during treatment, but they should not be allowed to displace direct assessment of symptoms and function (Spencer et al. 2026). Until such work is prospectively validated, symptom‐based and person‐centred assessment remains the most credible foundation for practice.

Within the proposed framework, symptom elicitation, administration of the brief core assessment, longitudinal documentation, recognition of discordant trajectories, reinforcement of agreed skin‐care measures and communication of unresolved burden can be led by the kidney nurse within local scope of practice. Diagnostic exclusion of alternative causes, initiation or modification of pharmacological therapy, and management of complex dermatological, neurological, psychiatric or sleep presentations remain prescriber‐led or multidisciplinary decisions. This separation is consistent with contemporary dialysis symptom‐management guidance and may support structured monitoring without blurring professional responsibilities (Bennett et al. 2025; Liossatou et al. 2025).

### 4.1 Implications for Clinical Practice

Response assessment can combine itch intensity with sleep, daily functioning and wider symptom burden (Weiner et al. 2024; Ständer et al. 2024; Bai et al. 2025; Bennett et al. 2025). Table 2 distinguishes a proposed core set from extended assessment. The proposed core contains four patient‐reported items across three domains: WI‐NRS for the worst itch during the past 24 h, using its established 0–10 anchors; two sleep prompts—‘During the past 7 days, how much has itching interfered with your sleep?’ (0, *not at all*; 10, *completely*) and ‘During the past 7 days, on how many nights did itching wake you or make it difficult to return to sleep?’ (0–7 nights); and one daily‐burden prompt—‘During the past 7 days, how much has itching interfered with your usual daily activities?’ (0, *not at all*; 10, *completely*). The three burden prompts and the combined core set are unvalidated proposals, with no established meaningful‐change thresholds or composite score. Repeated assessments should retain each item's wording, anchors and recall period. Skindex‐10 and 5‐D Itch can provide extended assessment using their established instructions (Vernon et al. 2026).

**Table 2:** Proposed core and extended assessment domains for monitoring of difelikefalin response.

Domain	Level	Suggested assessment	Suggested timing/frequency	Interpretation	Action if burden persists
Itch intensity	Core	WI‐NRS, retaining its established 0–10 anchors and 24‐h recall period	Proposed: baseline; around Weeks 4 and 12; then every 8–12 weeks when clinically stable, or earlier after symptom change. The optimal schedule is unvalidated.	A ≥ 3‐point reduction has psychometric support as a conservative meaningful within‐patient change threshold in trial populations with moderate‐to‐severe CKD‐aP, using weekly means of daily ratings. Application to isolated chairside ratings is an extrapolation; smaller changes may still matter to an individual (Vernon et al. 2021).	Confirm treatment exposure and diagnosis; review competing contributors and communicate persistent burden to the prescriber
Sleep‐related disruption	Core	Two author‐proposed prompts: itch‐related sleep interference (0–10) and nights disrupted by itch (0–7), both referring to the past 7 days	At the same core response checkpoints	Unvalidated monitoring prompts with no established meaningful‐change thresholds; interpret within‐person change and its importance to the person receiving care	Assess nocturnal itch and other sleep contributors; consider targeted sleep, mental health or medical review as appropriate
Day‐to‐day burden	Core	Standardised pragmatic question: ‘During the past 7 days, how much has itching interfered with your usual daily activities?’ rated from 0 (*not at all*) to 10 (*completely*)	At the same core response checkpoints	Author‐proposed prompt with no established meaningful‐change threshold; interpret longitudinal change and the residual problem prioritised by the person receiving care	Identify the residual domain that matters most and assign a specific action or escalation
Pruritus‐related quality of life	Extended	Skindex‐10 and/or 5‐D Itch scale	Baseline and Week 12; repeat when response is uncertain, discordant or clinically changed	A ≥ 15‐point reduction in Skindex‐10 and ≥ 5‐point reduction in 5‐D Itch represent meaningful within‐patient change (Vernon et al. 2026)	Persistent impairment despite itch improvement may be described as partial or discordant response within the proposed, unvalidated communication scheme; reassess the residual domain.
Associated symptom burden	Extended/triggered	Fatigue, pain, anxiety, low mood and other coexisting symptoms; use a locally adopted validated symptom instrument when broader assessment is needed	Baseline when burden is multidimensional; thereafter according to symptom severity, discordance and local workflow	Interpret longitudinally; these measures are not difelikefalin‐specific response thresholds	Route the dominant residual symptom to the appropriate member of the kidney care team
Broader clinical review	Triggered	Dry skin, primary skin disease, neuropathic features, medication changes, dialysis‐related factors, mineral and bone disorder and other systemic causes of itch	Whenever response is partial, absent or worsening	Not a treatment‐response score; identifies potentially modifiable competing contributors	Reinforce skin care and pursue prescriber, dermatology, neurology, mental health or other specialist review when indicated

*Note:* The proposed core set comprises four patient‐reported items across three domains: WI‐NRS, two sleep prompts and one daily‐burden prompt. The combined set, three burden prompts, within‐session assessment timing and follow‐up schedule—including reviews around Weeks 4 and 12 and subsequent 8‐ to 12‐week intervals—are unvalidated implementation proposals. Full response, partial response and persistent burden are pragmatic communication labels, not validated treatment‐response phenotypes. Instrument‐specific thresholds do not validate these labels or the combined pathway. No composite score or validated classification cut‐offs are proposed.

Abbreviations: CKD‐aP, chronic kidney disease‐associated pruritus; 5‐D Itch, 5‐D Itch scale; WI‐NRS, Worst Itching Intensity Numerical Rating Scale

The timing of symptom assessment within a haemodialysis session has not been established. As an unvalidated implementation proposal, brief measures could be administered at a consistent session phase, after treatment‐start procedures and before post‐dialysis difelikefalin administration, while avoiding acute intradialytic symptoms or end‐of‐session fatigue. This concerns assessment timing, not a change in drug administration. Baseline assessment would precede or coincide with treatment initiation. We propose an early review around Week 4 and a multidimensional review around Week 12, informed by trial assessment points (Fishbane et al. 2020; Zuo et al. 2026). Trial use of these time points does not validate this clinical review schedule. For clinically stable adults, subsequent review every 8–12 weeks is a pragmatic proposal; earlier review may be needed after symptom change. The optimal interval and the feasibility and clinical utility of the schedule require prospective evaluation.

The proposed four‐item core set is intended to fit routine haemodialysis care, but its completion time and feasibility have not been tested. Services evaluating it should audit completion time, missing responses and acceptability. Extended instruments could be reserved for baseline characterisation, planned multidimensional review, uncertain response or discordant domains. Documentation could use existing nursing or multidisciplinary records to capture baseline and current scores, residual burden prioritised by the person receiving care, the provisional communication label, planned action, responsible team member and reassessment date. Instrument‐specific thresholds and unresolved concerns can inform review, without operating as automatic treatment rules (Mehrotra et al. 2023; Bennett et al. 2025).

A practical review of residual burden is equally important. If itch improves but sleep remains poor, quality of life remains impaired, or symptom clusters remain prominent, then treatment should not automatically be judged a complete success; within the proposed communication scheme, this may be described as a partial or discordant response requiring domain‐specific reassessment. Likewise, persistent burden should prompt review of dry skin care, neuropathic features, dialysis adequacy, comorbid symptom distress and other possible contributors rather than immediate assumptions about treatment failure alone (Buades et al. 2024; Verduzco and Shirazian 2020; Latus et al. 2025; Kljajić et al. 2026).

Persistent nocturnal burden should prompt assessment of sleep disruption and competing contributors; prominent xerosis should prompt reinforcement of skin care; neuropathic features or atypical localisation should prompt diagnostic reassessment; and persistent or worsening itch despite adequate treatment exposure should be communicated to the prescriber for review of diagnosis, treatment strategy and other causes. Fatigue, anxiety, low mood or pain that remains clinically important should be routed to the appropriate member of the kidney care team rather than being attributed automatically to pruritus. The response labels alone should not determine continuation, switching or discontinuation of treatment.

Finally, routine assessment should be explicitly person‐centred. Kidney care professionals should ask not only whether itch is less severe, but also whether the person is sleeping better, scratching less at night, feeling less distressed and experiencing less interference with daily life. This emphasis aligns treatment evaluation with outcomes that adults receiving haemodialysis experience directly and that matter in everyday care (Rhee et al. 2022; Sukul et al. 2023; Wang et al. 2025).

Assessment should also remain accessible to adults with limited health literacy, language barriers or cognitive difficulties. Whenever possible, the same simple wording and response anchors should be used longitudinally. Questions may be staff‐administered rather than self‐completed, and visual 0–10 response scales can reduce reading burden. Validated translated versions of instruments should be preferred where available, with professional interpreter support when required. Cognitive impairment should be documented because it may affect the reliability of serial self‐report; proxy observations, when necessary, should be identified as such and should not automatically be treated as equivalent to patient‐reported symptom intensity (Mehrotra et al. 2023; Bennett et al. 2025).

### 4.2 Limitations

This review has several limitations. First, it is a structured narrative review rather than a systematic review, and study selection therefore remains susceptible to selection bias and author judgement despite a transparent search and eligibility framework. Second, no formal study‐level risk‐of‐bias assessment or quantitative evidence grading was undertaken, and studies of heterogeneous design were synthesised qualitatively. Third, the evidence base is dominated by adults receiving maintenance haemodialysis and should not be extrapolated directly to peritoneal dialysis, non‐dialysis CKD or paediatric populations. Fourth, evidence supporting meaningful change is stronger for the WI‐NRS, Skindex‐10 and 5‐D Itch scale than for the proposed brief sleep and daily‐burden questions; the latter are pragmatic monitoring prompts rather than validated standalone instruments. Fifth, the combined four‐item core set, brief burden questions and communication labels have not undergone prospective validation. Within‐session assessment timing, reviews around Weeks 4 and 12, and subsequent 8‐ to 12‐week intervals are also unvalidated implementation proposals. Feasibility, completion time, acceptability, consistency of category assignment and clinical utility require prospective evaluation. Applying the WI‐NRS change threshold to isolated chairside ratings also extrapolates beyond the weekly averages used in its validation. Finally, health literacy, language, culture and cognitive impairment may affect the feasibility and reliability of patient‐reported assessment, and several pivotal difelikefalin and psychometric studies were industry‐sponsored. These limitations reinforce the need for prospective evaluation of the proposed framework before it is regarded as a validated standard of care.

## 5 Conclusion

Evidence supports multidimensional assessment of difelikefalin response in adults receiving haemodialysis, incorporating itch, sleep, pruritus‐related quality of life and residual symptom burden (Weiner et al. 2024; Ständer et al. 2024; Bai et al. 2025; Bennett et al. 2025). We propose a person‐centred framework to organise this assessment. Its combined four‐item core set, brief burden questions, response labels, within‐session assessment timing and follow‐up schedule—including later 8‐ to 12‐week intervals—are pragmatic proposals requiring prospective evaluation. Validation of individual instruments does not establish validity or clinical benefit of the combined pathway. Exploratory biomarkers remain research tools and should not guide routine treatment decisions (Spencer et al. 2026). Prospective studies should assess feasibility, acceptability, consistency of interpretation and clinical utility before the framework is considered a validated clinical pathway.

## Author Contributions

**Lucas Maciel de Almeida Corrêa:** conceptualisation, literature review, writing – original draft, writing – review and editing. **Isabela Malerba Pinheiro:** literature review, writing – review and editing. **Yan Roberth Delmiro Silva:** literature review, writing – review and editing. **Guilherme Diniz Ferreira:** supervision, critical revision, writing – review and editing.

## Funding

The authors have nothing to report.

## Conflicts of Interest

The authors declare no conflicts of interest.

## Supporting information


Supporting File

## Data Availability

Data sharing is not applicable to this article as no data sets were generated or analysed during the current study.
