# Supplementary material for: Assessing Response to Difelikefalin in Adults Receiving Haemodialysis: A Multidisciplinary, Person‐Centred Framework for Chronic Kidney Disease‐Associated Pruritus
Source: J Ren Care. 2026 Nov 11;52(4):e70091. doi: 10.1111/jorc.70091 (PMC13753879; doi:10.1111/jorc.70091)
Supplement: Supplementary file 1 [file JORC-52-0-s001.docx]

**Supplementary Table S1. Database-specific search strategies used for the structured pragmatic literature search**

Two complementary searches were used: (A) difelikefalin/treatment-response evidence and (B) multidimensional assessment/implementation evidence. Search syntax was adapted to the indexing conventions of each database.

| **Database** | **Search concept** | **Exact search strategy** |
| --- | --- | --- |
| **PubMed** | A. Difelikefalin / treatment response | ((difelikefalin[Title/Abstract] OR CR845[Title/Abstract]) AND ("chronic kidney disease-associated pruritus"[Title/Abstract] OR "CKD-associated pruritus"[Title/Abstract] OR CKD-aP[Title/Abstract] OR "uraemic pruritus"[Title/Abstract] OR "uremic pruritus"[Title/Abstract] OR pruritus[Title/Abstract] OR itch[Title/Abstract]) AND (haemodialysis[Title/Abstract] OR hemodialysis[Title/Abstract] OR dialysis[Title/Abstract])) |
| **PubMed** | B. Multidimensional assessment / implementation | (("chronic kidney disease-associated pruritus"[Title/Abstract] OR "CKD-associated pruritus"[Title/Abstract] OR CKD-aP[Title/Abstract] OR "uraemic pruritus"[Title/Abstract] OR "uremic pruritus"[Title/Abstract]) AND (haemodialysis[Title/Abstract] OR hemodialysis[Title/Abstract] OR dialysis[Title/Abstract]) AND ("patient-reported outcome"[Title/Abstract] OR "patient-reported outcomes"[Title/Abstract] OR "patient reported outcome"[Title/Abstract] OR "patient reported outcomes"[Title/Abstract] OR PROM[Title/Abstract] OR PROMs[Title/Abstract] OR "symptom burden"[Title/Abstract] OR sleep[Title/Abstract] OR "quality of life"[Title/Abstract] OR Skindex[Title/Abstract] OR "5-D Itch"[Title/Abstract] OR WI-NRS[Title/Abstract] OR nurs*[Title/Abstract] OR implement*[Title/Abstract] OR biomarker*[Title/Abstract] OR "response assessment"[Title/Abstract])) |
| **Scopus** | A. Difelikefalin / treatment response | TITLE-ABS-KEY(difelikefalin OR CR845) AND TITLE-ABS-KEY("chronic kidney disease-associated pruritus" OR "CKD-associated pruritus" OR "CKD-aP" OR "uraemic pruritus" OR "uremic pruritus" OR pruritus OR itch) AND TITLE-ABS-KEY(haemodialysis OR hemodialysis OR dialysis) |
| **Scopus** | B. Multidimensional assessment / implementation | TITLE-ABS-KEY("chronic kidney disease-associated pruritus" OR "CKD-associated pruritus" OR "CKD-aP" OR "uraemic pruritus" OR "uremic pruritus") AND TITLE-ABS-KEY(haemodialysis OR hemodialysis OR dialysis) AND TITLE-ABS-KEY("patient-reported outcome" OR "patient-reported outcomes" OR "patient reported outcome" OR "patient reported outcomes" OR PROM OR PROMs OR "symptom burden" OR sleep OR "quality of life" OR Skindex OR "5-D Itch" OR WI-NRS OR nurs* OR implement* OR biomarker* OR "response assessment") |
| **Web of Science Core Collection** | A. Difelikefalin / treatment response | TS=(difelikefalin OR CR845) AND TS=("chronic kidney disease-associated pruritus" OR "CKD-associated pruritus" OR "CKD-aP" OR "uraemic pruritus" OR "uremic pruritus" OR pruritus OR itch) AND TS=(haemodialysis OR hemodialysis OR dialysis) |
| **Web of Science Core Collection** | B. Multidimensional assessment / implementation | TS=("chronic kidney disease-associated pruritus" OR "CKD-associated pruritus" OR "CKD-aP" OR "uraemic pruritus" OR "uremic pruritus") AND TS=(haemodialysis OR hemodialysis OR dialysis) AND TS=("patient-reported outcome" OR "patient-reported outcomes" OR "patient reported outcome" OR "patient reported outcomes" OR PROM OR PROMs OR "symptom burden" OR sleep OR "quality of life" OR Skindex OR "5-D Itch" OR WI-NRS OR nurs* OR implement* OR biomarker* OR "response assessment") |
| **Embase (Embase.com)** | A. Difelikefalin / treatment response | (difelikefalin:ti,ab,kw OR CR845:ti,ab,kw) AND ('chronic kidney disease-associated pruritus':ti,ab,kw OR 'CKD-associated pruritus':ti,ab,kw OR 'CKD-aP':ti,ab,kw OR 'uraemic pruritus':ti,ab,kw OR 'uremic pruritus':ti,ab,kw OR pruritus:ti,ab,kw OR itch:ti,ab,kw) AND (haemodialysis:ti,ab,kw OR hemodialysis:ti,ab,kw OR dialysis:ti,ab,kw) |
| **Embase (Embase.com)** | B. Multidimensional assessment / implementation | ('chronic kidney disease-associated pruritus':ti,ab,kw OR 'CKD-associated pruritus':ti,ab,kw OR 'CKD-aP':ti,ab,kw OR 'uraemic pruritus':ti,ab,kw OR 'uremic pruritus':ti,ab,kw) AND (haemodialysis:ti,ab,kw OR hemodialysis:ti,ab,kw OR dialysis:ti,ab,kw) AND ('patient-reported outcome':ti,ab,kw OR 'patient-reported outcomes':ti,ab,kw OR 'patient reported outcome':ti,ab,kw OR 'patient reported outcomes':ti,ab,kw OR PROM:ti,ab,kw OR PROMs:ti,ab,kw OR 'symptom burden':ti,ab,kw OR sleep:ti,ab,kw OR 'quality of life':ti,ab,kw OR Skindex:ti,ab,kw OR '5-D Itch':ti,ab,kw OR WI-NRS:ti,ab,kw OR nurs*:ti,ab,kw OR implement*:ti,ab,kw OR biomarker*:ti,ab,kw OR 'response assessment':ti,ab,kw) |

**Eligibility limits applied during selection:** English-language publications published from 1 January 2020 through 15 September 2026. No study-design filter was applied to the database searches.

**Additional source identification:** Reference lists of key reviews and pivotal studies were checked for directly relevant publications. Selected professional-society guidance or educational resources were considered when directly relevant to implementation or role delineation; grey literature was not otherwise systematically searched, and conference abstracts were not used as formal evidentiary support.

**Abbreviations:** CKD-aP, chronic kidney disease-associated pruritus; PROM, patient-reported outcome measure; WI-NRS, Worst Itching Intensity Numerical Rating Scale.
